# Evaluation of the “3 Good Questions” program for shared decision-making in pediatric medicine: a feasibility study

**DOI:** 10.1007/s00431-020-03868-1

**Published:** 2020-11-09

**Authors:** Robyn Rexwinkel, Hester Rippen, Inge J. M. Blokzijl-Boezeman, Zonja de Klein, Christel M. Walhof, Josine van der Kraan, Marc A. Benninga, Merit M. Tabbers

**Affiliations:** 1 Emma Children’s Hospital, Amsterdam UMC, University of Amsterdam, Pediatric Gastroenterology, Room C2-312, PO Box 22700, 1100 DD Amsterdam, The Netherlands; 2 Dutch Child and Hospital Foundation, Utrecht, The Netherlands; 3 Department of Pediatrics, Bernhoven, Uden, The Netherlands; 4 Department of Pediatrics, Ommelander Hospital Groningen, Groningen, The Netherlands; 5 Department of Pediatrics, Rijnstate, Arnhem, The Netherlands; 6 Dutch Patient Federation, Utrecht, The Netherlands

**Keywords:** Children, Shared decision-making, 3 Good Questions, Intervention

## Abstract

The “3 Good Questions” program was developed to increase shared decision making. The current pilot-study determined the feasibility of these questions to increase shared decision-making in Dutch pediatric medicine. Pre-/postintervention surveys were used to include children (10–18 years) at pediatric outpatient clinics of four hospitals in the Netherlands. After their appointment, two different groups of children completed the questionnaires. Group 1 filled in the survey before the intervention; group 2 completed the survey after active implementation of the “3 Good Questions” program. The primary outcome was to determine the feasibility (reach, applicability). Secondary outcomes were related to patient involvement in healthcare and treatment decisions and decision-making process between child and healthcare professional. In total, 168 and 114 children in groups 1 and 2 (61 vs 63% female, *P* = 0.68; age 13.3 ± 2.4 vs 13.8 ± 2.4 years, *P* = 0.72), respectively, completed the questionnaire. In group 2, 44% of children were aware of the “3 Good Questions”, of whom 18% posed ≥ 1 of the “3 Good Questions” during their appointment (feasibility). The “3 Good Questions” program led to more shared decision-making (SDM-Q-9: *P* = < 0.001;95%CI: − 2.43 to − 1.17). The majority of children who have read or heard of the “3 Good Questions” would recommend this program to other children.

*Conclusion:* Implementation of the “3 Good Questions” program seemed feasible, although it is necessary to further explore the implementation of this program at national level as a simple way for children and healthcare professionals to share decisions in practice.

**Table:** 

**What is known** • *Children have the right to be included in decision-making, and inclusion can improve patient satisfaction and quality of care, and reduce costs.* • *The “3 Good Questions” program was successfully implemented in adult healthcare to increase shared decision making, and therefore these “3 Good Questions” have been adapted to a child version.* **What is new** • *In this pilot study, we found that the implementation of the “3 Good Questions” program to increase shared decision-making in pediatric medicine seemed feasible. Although it is necessary to further explore the implementation of the “3 Good Questions” program at national level as a simple way for children and healthcare professionals to share decisions in practice.*

**Supplementary Information:**

The online version contains supplementary material available at 10.1007/s00431-020-03868-1.

## Introduction

Shared decision making (SDM) is the process by which the optimal decision for diagnosis, treatment or follow-up may be reached for a patient when more than one medically reasonable option is available [1, 2]. SDM aims to encourage healthcare professionals (HCPs) to include patients in decision-making, to improve patient satisfaction and quality of care, and reduce costs [3–9].

SDM in pediatrics is different than SDM in adult medicine. Specifically, in pediatrics, the extent to which the patient can be involved in SDM varies significantly globally. In the Netherlands, legislation around SDM for children is recorded in the Dutch Medical Treatment Act (WGBO) [10]. According to the Dutch law, parents are the legal decision-makers in children < 12 years old, whereas children aged 12–15 years may be the primary decision-makers in their own care, and children 16 years of age or older are able to give their own consent. However, a systematic review found that pediatric SDM interventions mainly focus on parents and that children are often not involved in decisions [11]. This is in contrast to current recommendations that state that children (aged 4–18) should be encouraged and supported to participate in healthcare decisions [12, 13]. Earlier research in children with cancer showed that SDM interventions in pediatrics have favorable effects, improving parental knowledge, child’s satisfaction and reducing the risk of a decisional conflict [11, 13, 14]. Still, data on SDM in pediatrics are scarce and further intervention studies are necessary to increase SDM in pediatric medicine.

To promote SDM in adult healthcare, two interventions were successfully developed in Australia (“Ask. Share. Know”) and the UK (“Ask 3 Questions”) [15–18]. These interventions led to a higher uptake of question asking during consultations, strengthen patient–physician communication and improved quality and safety of patient care. Recently, this “3 Good Questions” intervention (3GQ) has been translated to Dutch and is successfully implemented in the Dutch adult population [19]. However, in the development of this intervention, children’s language level and comprehension have not been taken into account. Therefore, in 2016, the 3GQ have been adapted to a child version with the aim to improve the quality of information provided during patient–physician consultations and in facilitating patient involvement within pediatric care. The 3GQ were developed and validated by the Dutch Child and Hospital Foundation during four focus groups (Box 1) [20]. The primary aim of the current study was to determine the feasibility of this 3GQ program to increase SDM in Dutch pediatric medicine, and secondary outcomes were related to patient involvement in healthcare and treatment decisions and decision-making process between child and HCP.

**Table:** Box 1 The “3 Good Questions” for children

1. This is what I feel, what is it?’ 2. ‘What can we do about it?’ 3. ‘What does this mean for me now and later?	

## Methods

### Design, participants, and setting

**Fig. 1:**
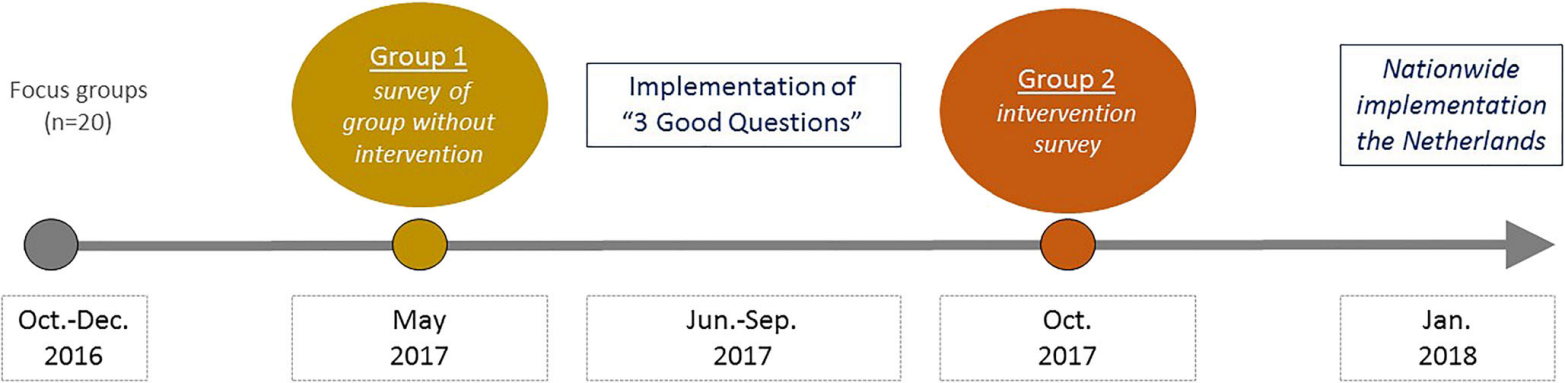
Flow diagram of the study

For this prospective pilot study, we used anonymous pre-/postintervention surveys, which were conducted at the pediatric gastroenterology outpatient clinic of one tertiary care hospital, (Amsterdam UMC) and at the general pediatric outpatient clinics of three secondary care hospitals in the Netherlands (Bernhoven, Ommelander, Rijnstate). Children were invited to participate in this survey by their treating physician if they were (1) 10–18 years of age; (2) attending an appointment at the pediatric outpatient clinic; and (3) were able to provide oral consent. Insufficient knowledge of the Dutch language was an exclusion criterion (e.g., in need of an interpreter). Detailed study information was provided by their treating physician. Two different groups of children completed the paper questionnaires. Group 1 fills out the questionnaire at baseline, when the 3GQ program was not implemented yet. Next, the 3GQ program was implemented in the hospitals. Group 2 completed the questionnaire after implementation of the 3GQ program. Questionnaires were handed out by their treating pediatrician and administered by the local researcher. Both parents and physician were not present when the questionnaire was filled in. No interaction was possible. Figure 1 shows an overview of the study process.

### Data collection methods and measurement instruments

Data collection took place between May and October 2017 and consisted of quantitative surveys. These surveys included questions regarding baseline and consultation characteristics, SDM process (the 9-item validated SDM-Q-9[21] and CollaboRATE [22]), the child’s perceived role in SDM (CPS [23]), and postintervention surveys (group 2) also included questions regarding their attitudes towards the 3GQ program. All questionnaires were adapted for children and validated before the start of the study by the Dutch Child and Hospital Foundation. No substantial adjustments were made. Data on the SDM-Q-9, CollaboRATE, and CPS were recoded according to the following methodologies:

- The SDM-Q-9 evaluates the SDM process from the child’s perspective and contains nine questions, each describing one step of the SDM process. The items are scored on a six-point Likert scale ranging from “completely disagree” to “completely agree”. Items are summed and transformed into a 0 to 100 score [21].
- The CollaboRATE is a three-item questionnaire to assess three core SDM tasks during a consultation. Responses to each item range from 0 (“no effort was made”) to 9 (“every effort was made”) and were scored in a binary way; children who responded to all questions with a “9” were considered to have experienced SDM, and all others were not. The proportion of children who reported a score of 9 on each of the three CollaboRATE questions was calculated [22].
- The CPS was used to evaluate children’s preference for participation in SDM process. This scale used five statements to indicate different response categories that describe how the children want to be involved in SDM. On basis of their responses, children were categorized as “active” (“I prefer to make the final decision” or “I prefer to make the final decision after seriously considering my parents’/physician’s opinion”), collaborative (“I prefer that my parents/physician and I share responsibility for the decision”), or passive (“I prefer that my parents/physician make the decision after he/she seriously considers my opinion” or “I prefer my parents/physician to make the decision”) role. The CPS was asked once (combined focus on SDM with parents and with physicians) [23].

### Strategies to implement the 3GQ program

Implementation strategies were embedded in standard care as much as possible. For the 3GQ program, a multicomponent intervention was designed, consisting of four elements:

1. Information brochures were sent to the children’s home before the consultation, together with the confirmation letter for the appointment (Online Resource 1A).
2. Posters and pocket-sized cards were presented in the consultation rooms (Online Resource 1B).
3. Information was presented on digital screens and posters in waiting rooms, and incorporated a consultation summary sheet and website information (www.3goedevragen.nl/kinderen).
4. Patient organizations (Dutch Patient Federation (Patiëntenfederatie Nederland) published the information on their websites, blogs, newsletters, magazines, and social media.

### Primary and secondary outcomes

The primary outcome was to determine the feasibility of the 3GQ program in pediatric secondary and tertiary care, consisting of the percentage of children who had already heard of the 3GQ program (reach), and the extent to which the 3GQ program was used in healthcare consultations (applicability). Secondary outcome measures were measures related to patient involvement in healthcare and treatment decisions and decision-making process between child and HCP, as measured with the CollaboRATE, the Control Preferences Scale (CPS), and the Shared Decision Making Questionnaire (SDM-Q-9) [21–23].

### Statistical analysis

Frequency distributions and descriptive statistics were computed for survey questions. Independent *t* tests and Mann–Whitney *U* tests for continuous data and chi-square and Fisher’s extract tests for dichotomous data were used to test for differences between the two groups. For calculation of CollaboRATE and SDM-Q-9 scores, cases where responses on one or more of the items were missing were excluded. A significance level of 0.05 was used to define statistical significance. Statistical analysis was performed using IBM SPSS Statistics 25 (Chicago, IL, USA).

## Results

### Participant and consultation characteristics

In total, 168 and 114 children in group 1 and 2 (61 vs 63% female; age 13.3 ± 2.43 vs 13.8 ± 2.47 years), respectively, completed the questionnaire. In 69 vs 75% of the consultations, only the mother was present, following by both parents (15 vs 16%) and by only the father (12 vs 7%). The minority of children visited their HCP for the first time (18 vs 22%). Most of the children had an appointment with their own pediatrician. The duration of the consultation varied from 3 to 60 min. A number of different decisions were made during the consultations of which the most frequently decisions were a follow-up appointment (60 vs 55%) and diagnostic testing (24 vs 25%). All differences were not statistically significant between both groups. Characteristics of the study sample and consultations are shown in Table 1.

**Table 1:** Baseline characteristics of study sample (*n* = 282) and consultations

	Group 1 (*n* = 168 )	Group 2 (*n* = 114)	*P* value*
Baseline characteristics			
Gender (*n*, female %)	102 (60)	72 (63)	0.68
Age (mean, SD)	13.3 (2.4)	13.8 (2.5)	0.72
Hospital (*n*, %)			0.84
AMC Bernhoven Ommelander Rijnstate	43 (25) 32 (19) 67 (40) 26 (15)	27 (24) 26 (23) 46 (40) 15 (13)	
Present during the consultation (*n*, %):			0.47
Mother Both parents Father Alone Grandmother Aunt	116 (69) 25 (15) 21 (12) 4 (2) 2 (1) 1 (1)	85 (75) 18 (16) 8 (7) 3 (2) - -	
First appointment: yes (*n*, %)	31 (18)	25 (22)	0.47
Appointment with (*n*, %):			
Pediatrician Nurse Patient does not know Other (not further specified)	150 (89) 21 (12) 3 (2) 3 (2)	107 (94) 9 (8) 3 (3) 5 (4)	0.19 0.22 0.63 0.26
Duration (in min) (median, IQR)	15 (13-22.5)	20 (15-30)	0.11
Decision on next step (*n*, %)	159 (94)	105 (92)	0.39
What kind of decision was made (*n*, %):			
Follow-up appointment Diagnostic testing Start treatment Referral to other physician Adjust treatment Stop treatment	102 (60) 41 (24) 24 (14) 12 (7) 10 (6) 6 (4)	63 (55) 29 (25) 16 (14) 15 (13) 11 (10) 6 (5)	0.36 0.84 0.95 0.09 0.25 0.49

*P values as determined with χ^2^ tests, Fisher’s exact test, Mann–Whitney *U* test and unpaired *t* test

### Feasibility

Seventeen percent of the children in group 1 vs 25% in group 2, respectively, prepared (any) questions before their consultation (*P* = 0.09), and more than 95% actually asked these prepared questions during consultation (group 1:100 vs group 2:95%, *P* = 0.15). Furthermore, almost all children reported that they were encouraged to ask questions (group 1:92 vs group 2:93%, *P* = 0.76) and 91% in group 1 vs 93% in group 2, respectively, had the feeling their questions were sufficiently answered (*P* = 0.52).

In group 2, the intervention survey, 50/114 children (44%) receiving the 3GQ indicated to have heard of the 3GQ or have read them (reach). Of these children, 17/50 (34%) indicated that they prepared their appointment as a consequence differently. Nine out of these fifty children (18%) posed at least one of the questions during their appointment. Children (*n* = 50) agreed that the 3GQ helped them to get information (42%) and to discuss treatment options (40%) with their HCP. Forty-five out of the fifty (90%) children reported that their physician was prepared for the 3GQ.

Children were asked whether they had a healthcare decision to make during their consultation, and this information was compared to whether they asked at least one of the 3GQ. Of the 99/114 children who reported of having made a decision, 9 (9%) asked at least one of the 3GQ. In comparison, children who had no decision to make (*n* = 6), none asked one of the 3GQ.

Fifty children rated the components of the 3GQ program (brochure, poster, and pocket-sized card) as very useful. Twenty-eight out of fifty (56%) children were positive about the poster, 30/50 (60%) about the brochure, and 21/50 (42%) about the pocket-sized card. The majority of these fifty children (68%) reported that they would recommend the 3GQ program to other children.

### Effects of the 3GQ program on SDM during healthcare consultations

Perceived participation (SDM-Q-9) was relatively high both in group 1 (79.73 ± 2.72) and group 2 (81.53 ± 2.46). Compared to group 1, the group without intervention, children receiving the 3GQ program reported to be more involved in SDM (*P* = < 0.001; 95% CI: − 2.43 to − 1.17).

Overall, 44.0% in group 1 and 44.8% of the children in group 2 reported a maximal CollaboRATE score on the three aspects of SDM. This difference was not significant. Scores of the CollaboRATE are shown in Table 2.

**Table 2:** Scores on the CollaboRATE (%)

	Group 1 (*n* = 168)	Group 2 (*n* = 114)	*P* value*
How much effort was made to help you understand your health issues?	58.0	55.2	0.66
How much effort was made to listen to the things that matter most to you about your health issues?	55.4	60.0	0.46
How much effort was made to include what matters most to you in choosing what to do next?	56.7	56.2	0.94
Total ColaboRATE score	44.0	44.8	0.90

^a^The proportion of children who reported a score of 9 on each of the three CollaboRATE questions

*P values as determined with *χ*^2^ tests test

**Fig. 2:**
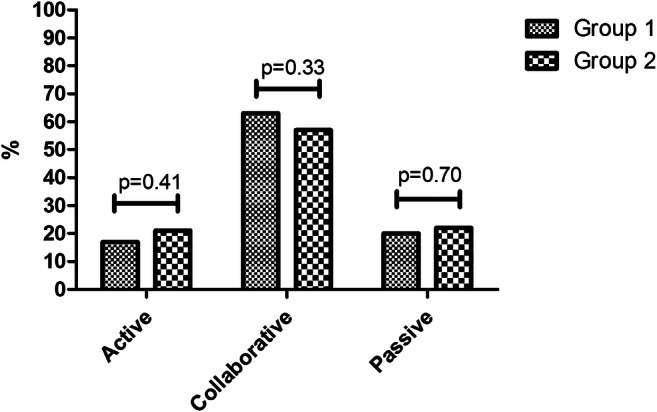
Comparing decision-making preferences for intervention and control group

A total of 264 out of 282 (94%) children completed the CPS questionnaire. Results in both groups showed that the majority of children perceived the decision-making process to be shared (collaborative role): 79 vs 77% (children < 12 years), 55 vs 49% (children 12–16 years) and 68 vs 57% (children > 16 years) (Table 3). When combining all age groups, most children also preferring a collaborative role in treatment decision-making (*P* = 0.33) (Fig. 2).

**Table 3:** Communication and decision preferences measured by the control preference scale (*n*, %)

	< 12 years		12–16 years		> 16 years	
	Group 1 (*n* = 38)	Group 2 (*n* = 22)	Group 1 (*n* = 93)	Group 2 (*n* = 53)	Group 1 (*n* = 28)	Group 2 (*n* = 30)
Active role	5 (13)	3 (14)	17 (18)	14 (26)	5 (18)	5 (17)
Collaborative role	30 (79)	17 (77)	51 (55)	26 (49)	19 (68)	17 (57)
Passive role	3 (8)	2 (9)	25 (27)	13 (25)	4 (14)	8 (27)

## Discussion

To the best of our knowledge, this is the first feasibility study evaluating the 3GQ program for children. Although we demonstrated that the brief 3GQ program created awareness towards the possibility for children to ask questions, only a few children used the 3GQ during consultation. The use of the 3GQ however led to more SDM between HCP and child, and was considered to be helpful to get information and to discuss treatment options with their HCP. The majority of children indicated to have heard of the 3GQ program would recommend the intervention to other children.

The 3GQ program was designed to assist children to make informed, as well as evidence-based, decisions. This study might show that children who asked the 3GQ were more likely to reach a decision in their consultation. This may suggests that the 3GQ program was useful in the decision-making process of these children. Our results are in line with other studies that report positive effects of interventions to support question asking and information provision and to improve participation in SDM. A metaanalysis that provided adult patients with an intervention before consultation to help patients address their information needs, also found an increase in the involvement of SDM [24]. The simplicity of the 3GQ program and the study findings suggest that a brochure, pocket-sized card, poster, and website, may be sufficient to promote patient engagement and facilitate SDM. Moreover, the large number of patients in this study that preferred an active or collaborative role in SDM, is in line with findings in the general population [25]. Especially for adolescents, it is important to be involved in decision-making, as a lack of it results in feelings of anger, inadequacy and frustration, and nonadherence with treatment [26].

Similar to other studies [19, 27, 28], we also found difficulties with the implementation of the 3GQ program in the pediatric population, as only 48% of the children receiving the 3GQ program had heard or read the questions prior to their appointment, of whom only 18% posed at least one of the questions during their appointment. This might be partly because not all children considered the 3GQ program to be necessary to get a more active role in SDM or to get more information. For example, it could be possible that (pediatric) HCPs already provide structured information during consultation, that children already asked the 3GQ by themselves during their consultation, or that pediatricians simply involve children more than other physicians in adult care by asking questions in order to have more discussion with the children concerning their disease. Also it could be possible that children only positively rated the 3GQ as “asked”, if they used the exact wording on the card, while it might be possible that almost similar questions are indeed asked during their consultation. Furthermore, the majority of children in this study had a follow-up appointment, whereas main decisions are frequently made during the first or second appointment. Moreover, in this pilot feasibility study, no implementation strategies for HCPs were included, while HCPs have an important role in SDM [29]. Including HCPs in this intervention seems important and should be taken into account when implementing the 3GQ program in the Netherlands. In contrast to the 3GQ program, studies in oncology successfully implemented decision-making interventions in adult cancer care [30, 31]. However, these interventions rely on the availability of trained coaches, do not exist for all clinical situations or health problems, take considerable time and resources to develop, and require regular updating as new evidence becomes available [32]. Therefore, the 3GQ program for children appears to fill a crucial gap in encouraging evidence-based SDM.

A systematic review in adults found an increase in consultation length as a result of the SDM interventions [24]. We think that HCPs might be concerned about this finding, yet it is not surprising when patients are encouraged to ask questions, because this leads to an increase in length of consultations. However, data analyzed from 17 studies concluded that SDM interventions do not lead to sizeable (10%) increases in length of consultations [33]. These findings are consistent with data obtained in this study as there was no significant difference in consultation length between the pre- and postintervention group. There are no studies undertaken which explored whether the time within the consultation was spent differently. Further research should be undertaken to investigate this important issue, as it may be as important as the amount of time itself [34].

Currently, there is no consensus regarding the optimum outcome measurement for SDM [35]. We therefore used multiple outcome measures to measure the extent of SDM practice from the perspectives of children, such as the SDM-Q-9 questionnaire, which is a strength of this study. The SDM-Q-9 is a generic instrument to appreciate the perceived level of SDM behavior, with an apparent celling effect [21]. There are several limitations in this study. First, we only included children, as parents and HCPs were left out of the study. Second, observational techniques were not used. In future research, qualitative interviews should receive more attention to identify barriers and facilitators of implementation [19]. The third limitation concerns to the study population. In our study, there are no data available on patients’ education, ethnicity, medical problems, and clinical characteristics, and only children from secondary and tertiary care hospitals were included. Therefore, the current results may not be generalizable in other settings. Earlier research showed that young Caucasian patients from the middle classes asked more questions compared to other groups [24]. This is an important issue for future research to identify those children in whom SDM interventions are plausible to be most beneficial. However, we performed our research in four hospitals in the Netherlands, reducing the risk of a nondiverse patient population. Fourth, different groups of patients completed the surveys. It is therefore possible that some (e.g., personal or disease-specific) characteristics influenced the decision-making process. Final, recall of the questions was low. Only a small sample size actually used the 3GQ during consultation.

Currently, HCPs increasingly acknowledge the importance of patient involvement. Unfortunately, there are still many HCPs who are not completely familiar with SDM or are not able to implement SDM due to limited consultation time. Previous research showed that HCPs rarely discuss treatment decisions with children [36]. A recent study found that not only children but also a trustful relationship between parents and HCP is helpful in child’s treatment [37], Parents mainly expect from HCP to give reassurance and answer their questions [38]. Therefore, targeted interventions are necessary. SDM training for HCPs might create consciousness, and consequently, improving SDM in the future. Since it takes three to tango, also parents and children need to engage in SDM [6]. Tools are available, such as digital decision aids, to assist parents and children in treatment decision by providing information about pros and cons of potential treatment options [39]. Moreover, it might be interesting to ask (“3 Good”) questions digitally before consultation, which might save time during consultations. If patients are encouraged routinely to establish the 3GQ for their HCPs before consultations, and if HCPs are routinely and effectively trained to set patients’ concerns, it is likely that both patients and HCPs will profit. In future research, the benefits of this 3GQ program on various financial and quality aspects of healthcare should be further investigated.

## Conclusion

The 3GQ program empowered children to ask the listed questions during consultations at the outpatient clinic, and has shown significant effects in improving the quality of information provided during consultations and in increasing SDM. While further evaluation to determine the generalizability of the study findings to other settings is needed, healthcare systems should proceed with implementing the 3GQ program at national level as a simple way for children and HCPs to share decisions in practice.

## Supplementary Information

Online Resource 1. The “3 Good Questions” poster (A) and brochure (B) (in Dutch) (PDF 2200 kb).

ESM 1 (PDF 670 kb).

## Data Availability

Not applicable.

## Abbreviations

3GQ: 3 Good Questions
CPS: Control preferences scale
HCP: Healthcare professional
SDM: Shared decision-making
SDM-Q-9: Shared decision-making questionnaire
