# Supplementary material for: Evaluation of the “3 Good Questions” program for shared decision-making in pediatric medicine: a feasibility study
Source: Eur J Pediatr. 2020 Nov 9;180(4):1235–42. doi: 10.1007/s00431-020-03868-1 (PMC7940148; doi:10.1007/s00431-020-03868-1)
Supplement: Supplementary file 1 — The “3 Good Questions” poster (A) and brochure (B) (in Dutch) (PDF 2200 kb). [file 431_2020_3868_MOESM1_ESM.pdf]

Stel deze drie goede vragen aan je dokter en beslis samen. Vul dit tijdens het gesprek met de dokter samen in:

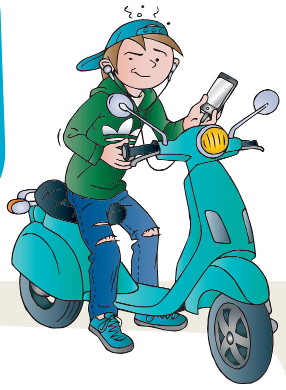

1. Dit voel ik, wat is het?

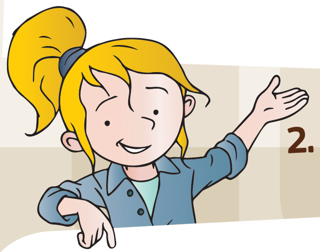

2. Wat kunnen we er allemaal aan doen?

3. Wat betekent dit voor mij nu en later?

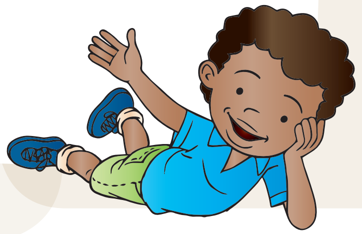

Meer weten?
www.3goedevragen.nl/kinderen

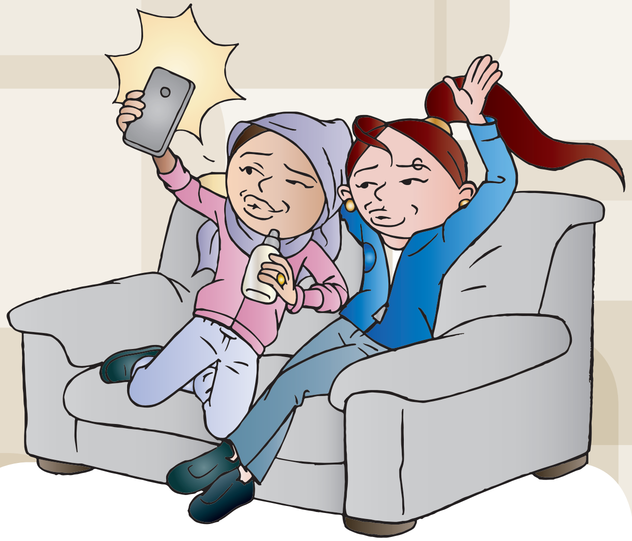

Initiatief van:

k&z kind&ziekenhuis

NVK Nederlandse Vereniging voor Kindergeneeskunde

Ben je nog geen 18 jaar en bij de dokter?
Je hebt altijd iets te kiezen. **Stel zelf ook**

Betere zorg begint met een goed gesprek

3 goede vragen

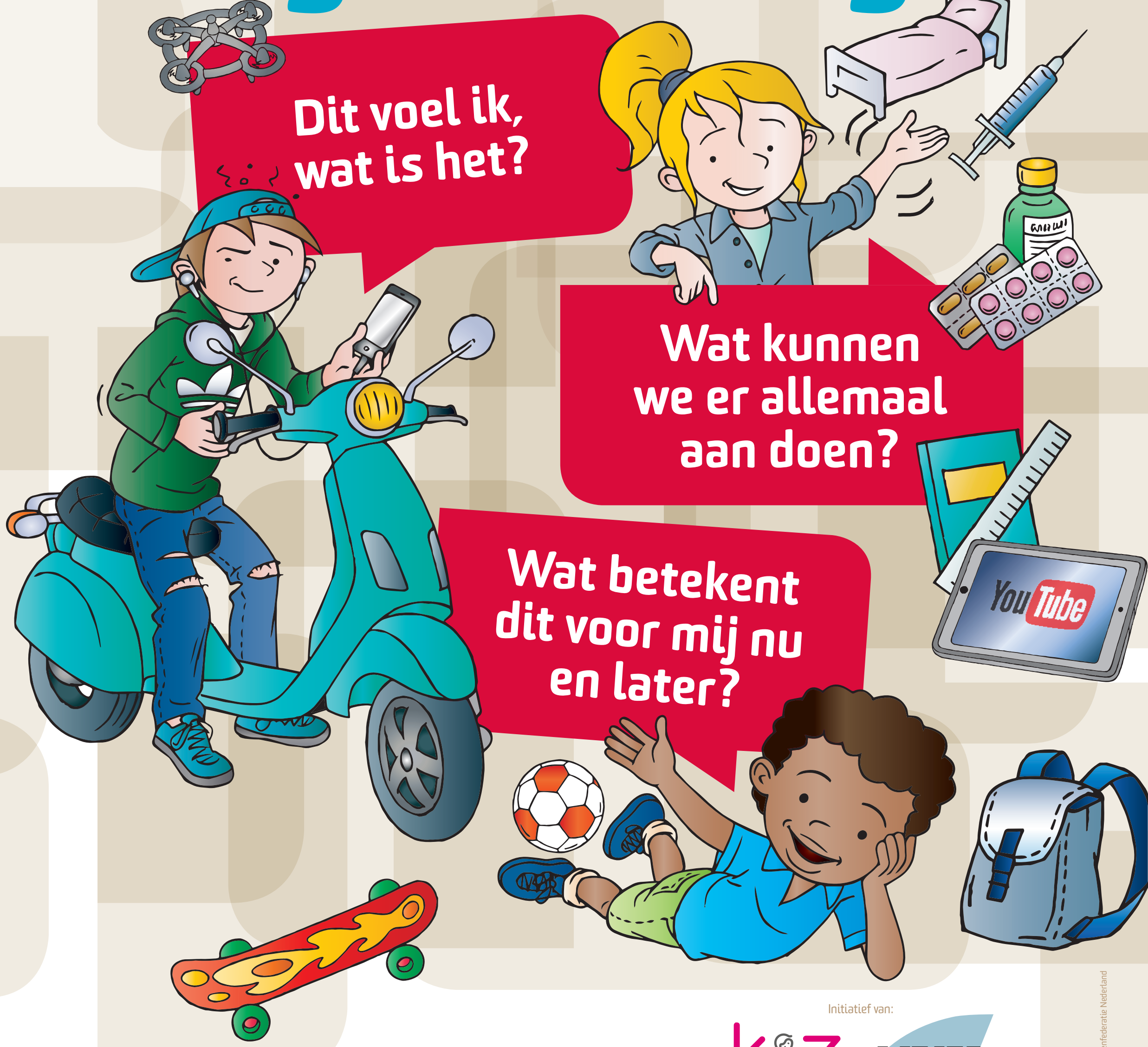

Meer weten?
www.3goedevragen.nl/kinderen

Initiatief van:

k&z kind&ziekenhuis

NVK Nederlandse Vereniging voor Kindergeneeskunde

© Patiëntenfederatie Nederland

Bij de dokter? Je hebt altijd iets te kiezen. Wat past het beste bij jou? Stel zelf ook drie goede vragen. Zo kan je samen met je ouder(s) en de dokter beslissen.

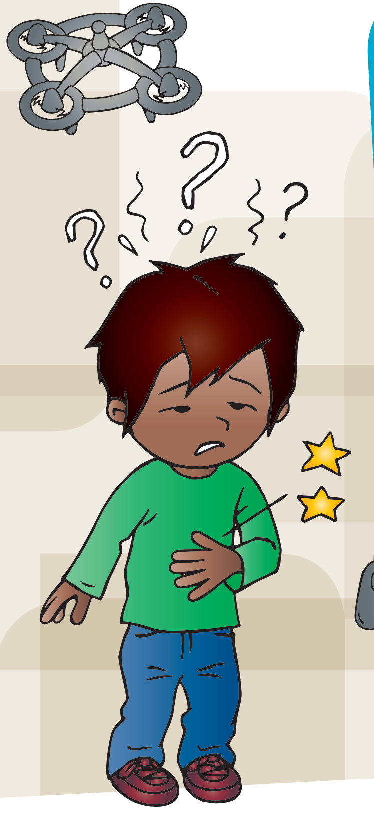

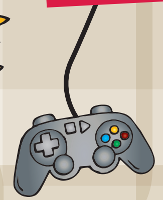

Zij stelden de drie vragen ...

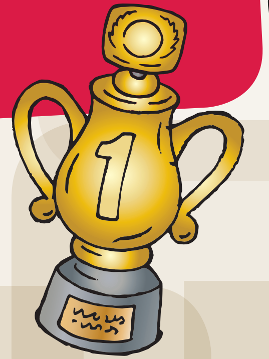

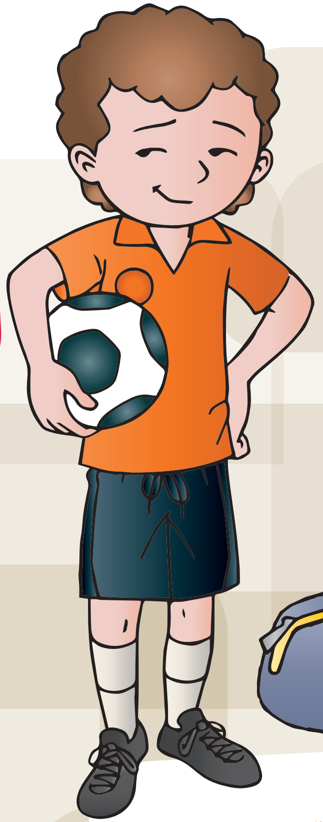

Meysam, 7 jaar: "Ik voel borrels in mijn buik en moet vaak naar de wc. Wat is het? Na enkele onderzoeken vertelde de dokter mij dat ik de ziekte van Crohn heb. **Ik mocht zelf vragen stellen aan de dokter, ik snap het nu veel beter**. De dokter legde uit wat we er allemaal aan konden doen".

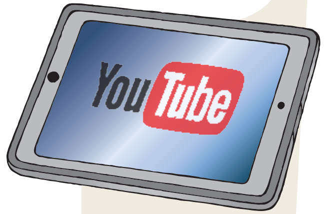

Sophie, 9 jaar: "Als ik aan het hockeyen ben, kan ik minder hard lopen dan eerst. Ik kan steeds minder goed ademen en krijg het benauwd. Ik heb dit toen tegen de dokter gezegd en toen hebben we ontdekt waar dit vandaan kwam. Ik blijk astma te hebben. Mijn vriendin heeft ook astma en die moest stoppen met hockeyen. Mijn dokter gaf aan dat **er meer opties zijn**. Ik wist niet dat ik zelf kon kiezen voor een andere behandeling. Gelukkig kan ik wel blijven hockeyen. Ik moet daarvoor wel mijn puffer extra gebruiken".

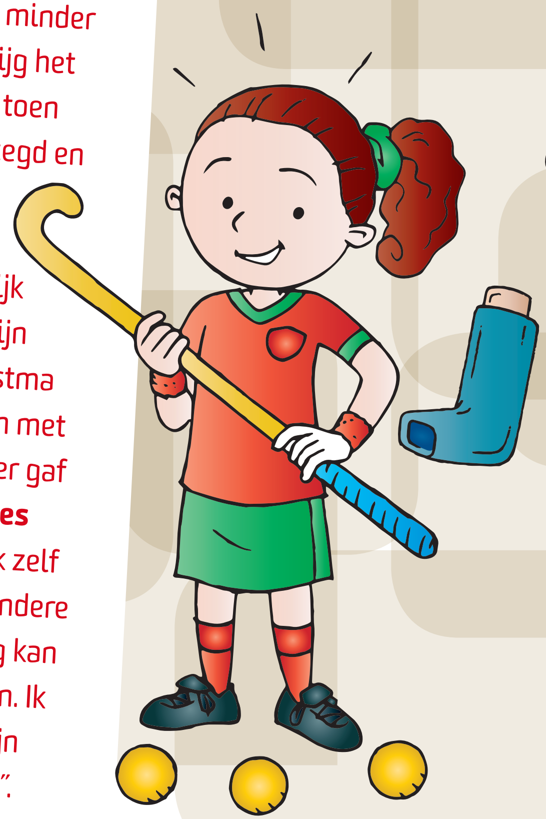

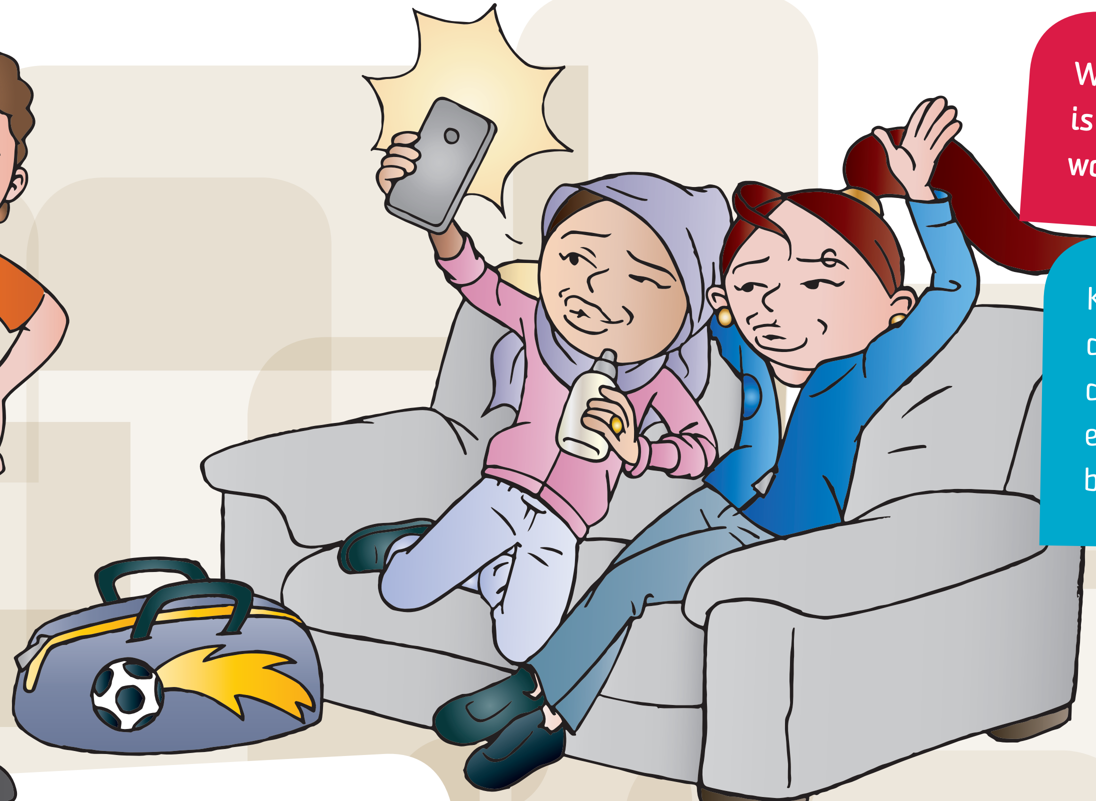

Bas, 16 jaar: "Elk voorjaar heb ik veel last van niezen en jeukende ogen. Dat wordt steeds langer en heftiger. Zit ik binnen terwijl mijn vrienden lekker buiten voetballen. Maar ik vergeet die pillen steeds en dan is het echt niet leuk om buiten te zijn. **De dokter legde me uit dat ik elke keer pilletjes en neusspray kan blijven gebruiken, maar dat ik ook 3 jaar lang elke maand 1 prik kan krijgen om zo helemaal van mijn allergie af te komen.** Dan zou ik weer zonder problemen buiten kunnen voetballen. Dat vind ik die prikken wel waard".

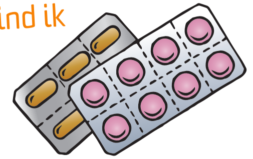

Marije, 16 jaar, tweelingzus van Bas: "Ook ik heb last van hooikoorts en pollenallergie. **Maar ik speel niet zo vaak buiten, zit meer met mijn vriendinnen te chillen**. Ik vind die pillen niet zo'n punt, ik vergeet ze ook nooit zoals Bas dat doet (die is zo slordig...), maar elke maand een spuit in mijn arm? No way, daar begin ik niet aan. Laat Bas dat maar doen. Voorlopig gaat het met mij goed zo."

Wie beslist er eigenlijk wat het beste voor je is als je bij de dokter komt? Moet er rekening worden gehouden met jouw mening?

Knappe koppen hebben een speciale wet gemaakt, de WGBO, waarin dat allemaal wordt geregeld. In die wet staat dat je altijd recht hebt op INFORMATIE en dat de dokter voor elk onderzoek en voor elke behandeling TOESTEMMING moet vragen.

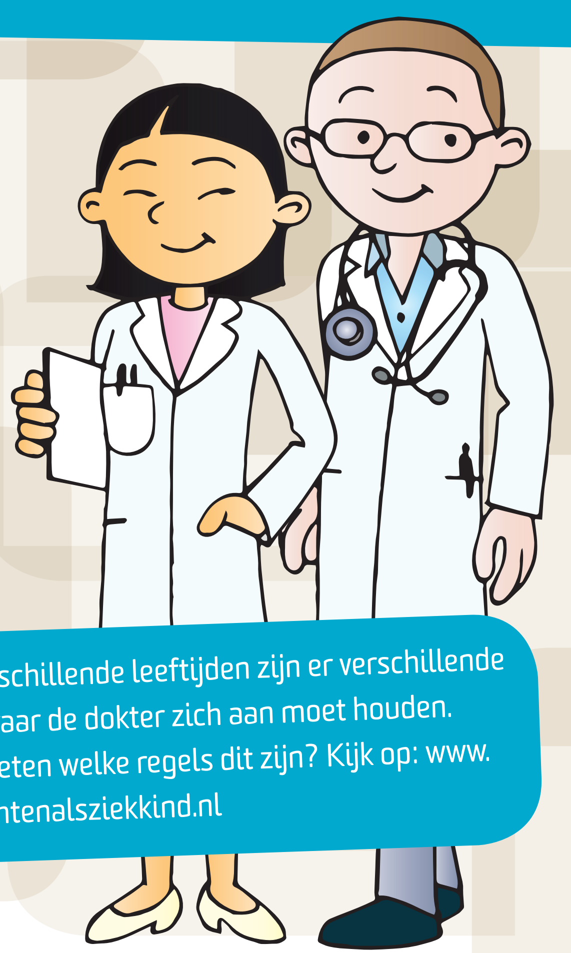

Voor verschillende leeftijden zijn er verschillende regels waar de dokter zich aan moet houden. Wil je weten welke regels dit zijn? Kijk op: www.mijnrechtenalsziekkind.nl
