# Supplementary material for: Evaluation of the “3 Good Questions” program for shared decision-making in pediatric medicine: a feasibility study
Source: Eur J Pediatr. 2020 Nov 9;180(4):1235–42. doi: 10.1007/s00431-020-03868-1 (PMC7940148; doi:10.1007/s00431-020-03868-1)
Supplement: Supplementary file 2 — (PDF 670 kb). [file 431_2020_3868_MOESM2_ESM.pdf]

Ben je nog geen 18 jaar en bij de dokter?
Je hebt altijd iets te kiezen. **Stel zelf ook**

Betere zorg begint met een goed gesprek

3 goede vragen

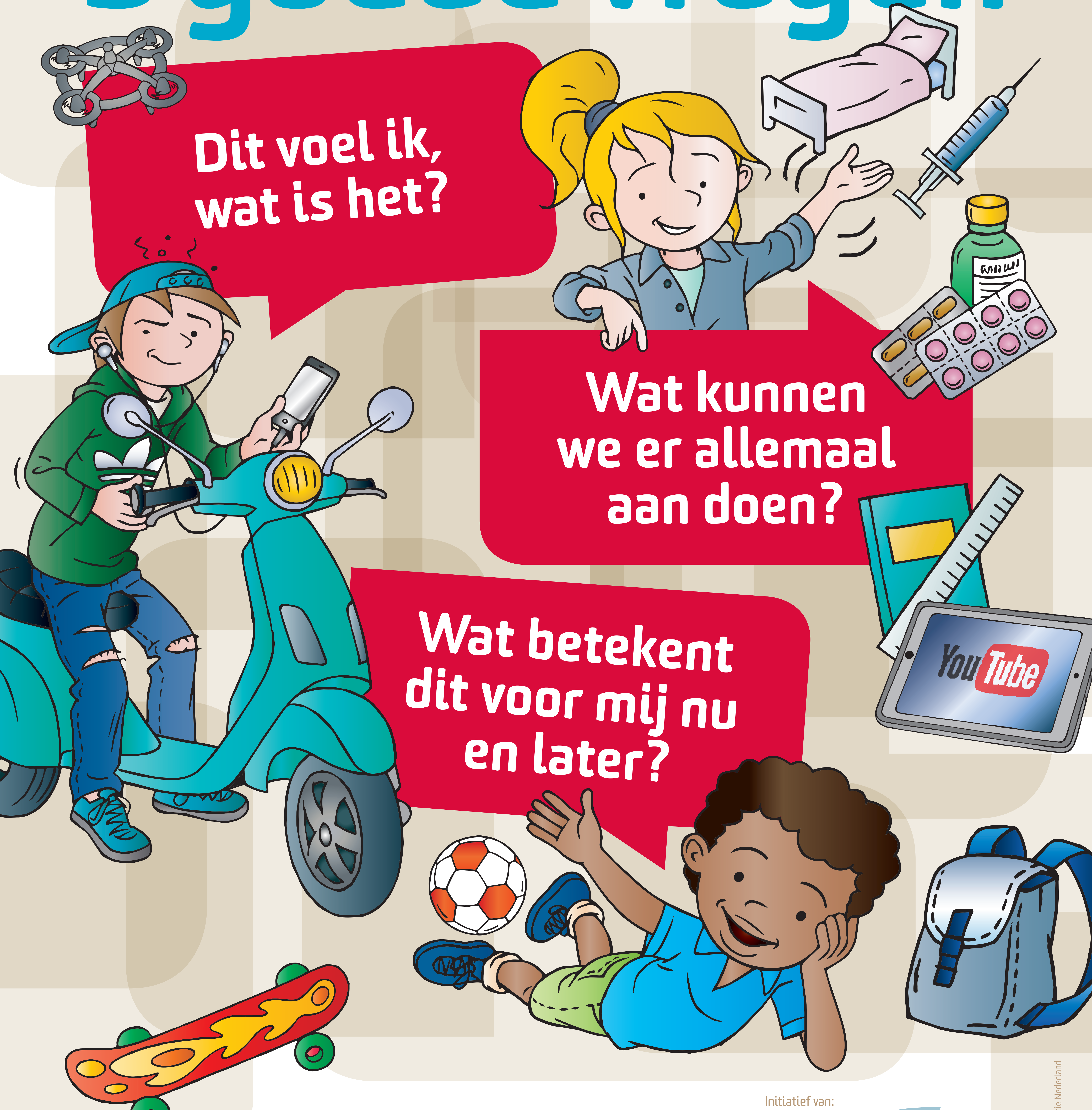

Dit voel ik, wat is het?

Wat kunnen we er allemaal aan doen?

Wat betekent dit voor mij nu en later?

Initiatief van:

k&z kind&ziekenhuis

NVK Nederlandse Vereniging voor Kindergeneeskunde

Meer weten?
www.3goedevragen.nl/kinderen

© Patiëntenfederatie Nederland
